# Compact and efficient O-band bismuth-doped phosphosilicate fiber amplifier for fiber-optic communications

**DOI:** 10.1038/s41598-020-68243-4

**Published:** 2020-07-09

**Authors:** Sergei V. Firstov, Aleksandr M. Khegai, Alexander V. Kharakhordin, Sergey V. Alyshev, Elena G. Firstova, Yan J. Ososkov, Mikhail A. Melkumov, Lyudmila D. Iskhakova, Elena B. Evlampieva, Alexey S. Lobanov, Mikhail V. Yashkov, Alexey N. Guryanov

**Affiliations:** 1 Prokhorov General Physics Institute of the Russian Academy of Sciences, Dianov Fiber Optics Research Center, Moscow, 119333 Russia; 2 G.G. Devyatyh Institute of Chemistry of High-Purity Substances of the Russian Academy of Sciences, Nizhny Novgorod, 603600 Russia; 3 MIREA-Russian Technological University, Moscow, 119454 Russia

**Keywords:** Fibre optics and optical communications, Fibre lasers

## Abstract

During last decades there has been considerable interest in developing a fiber amplifier for the 1.3-$\upmu$m spectral region that is comparable in performance to the Er-doped fiber amplifier operating near 1.55 $\upmu$m. It is due to the fact that most of the existing fiber-optic communication systems that dominate terrestrial networks could be used for the data transmission in O-band (1260–1360 nm), where dispersion compensation is not required, providing a low-cost increase of the capacity. In this regard, significant efforts of the research laboratories were initially directed towards the study of the praseodymium-doped fluoride fiber amplifier having high gain and output powers at the desired wavelengths. However, despite the fact that this type of amplifiers had rapidly appeared as a commercial amplifier prototype it did not receive widespread demand in the telecom industry because of its low efficiency. It stimulated the search of novel optical materials for this purpose. About 10 years ago, a new type of bismuth-doped active fibers was developed, which turned out to be a promising medium for amplification at 1.3 $\upmu$m. Here, we report on the development of a compact and efficient 20-dB (achieved for signal powers between $-40$ and $-10$ dBm) bismuth-doped fiber amplifier for a wavelength region of 1300–1350 nm in the forward, backward and bi-directional configurations, which can be pumped by a commercially available laser diode at 1230 nm with an output power of 250 mW. The compactness of the tested amplifier was provided by using a depressed cladding active fiber with low bending loss, which was coiled on a reel with a radius of 1.5 cm. We studied the gain and noise figure characteristics at different pump and signal powers. A record gain coefficient of 0.18 dB/mW (at the pump-to-signal power conversion efficiency of above 27$\%$) has been achieved.

## Introduction

In 2008, the first bismuth-doped fibers providing a net gain near 1.3 $\upmu$m were fabricated using the conventional MCVD technique^1^ that is considered as a starting point for more intensive research in this direction. Generally, these fibers had a very low efficiency ($< 5\%$) that was caused by the lack of data on the nature of the bismuth-related active centers (BACs) and the mechanisms of their formation, including the influence of the glass matrix and technological parameters^2^. Nevertheless, the first bismuth-doped phosphosilicate fiber amplifier (BDFA) operating in the range 1300–1340 nm was realized in 2010^3^. Using a unidirectional forward pumping configuration ($\lambda _{p}$ $=$ 1230 nm and $P_{p}$ $=$ 460 mW), a peak gain of 24.5 dB at 1320 nm with 3-dB bandwidth $\Delta \lambda \approx$ 37 nm and a minimum noise figure of 5 dB were achieved. By the virtue of further in-depth studies of the BACs responsible for the amplification at the wavelengths near 1300 nm, including inhomogeneous broadening effects^4,5^, along with the optimization of the production process^6^ and the chemical composition of the glass matrix^7^ it was possible to provide the improvement of the spectral characteristics of such fiber amplifiers. Also, the BDFA with an extended gain bandwidth (over a 40 nm) in the wavelength region between 1320 and 1360 nm has been constructed using a dual-wavelength pumping configuration^8^. In addition, it was shown that gain peak can be efficiently tuned in the O-band, which covers the wavelengths ranging from 1260 to 1360 nm, by a variation of the pump wavelength^9^. As a consequence, an interest in this type of the BDFA as a prospect device for the optical fiber access networks has been increasing. Recently, an experimental testing of this type of the BDFAs in fiber-optic communication systems operating in the O-band has been successfully performed^9–11^. Thereafter, the implementation of the O-band BDFAs may be considered as a simple way to upgrade the conventional 1.3 $\upmu$m fiber links. In this regard, the main task is the realization of a compact and efficient commercial prototype of the BDFA. Unfortunately, the length of the Bi-doped fibers required for the BDFA is usually more than 100 m because of the low gain per meter. Moreover, a rather low refractive index difference between the core and the cladding ($\Delta$n$\approx$0.004–0.005) of the Bi-doped fibers make them sufficiently sensitive to macrobending that prevents the winding of the fiber in a coil of a relatively small diameter (< 3 cm). Altogether these are key factors limiting the compactness of the BDFA. Furthermore, even at a very low active dopant content, this type of active fibers has a high level of the unsaturable loss (greater than 10$\%$ with respect to the active absorption), which negatively affects the efficiency of the device. For instance, to achieve gain of $>$ 20 dB, the required pump power is ranging from 460 to 800 mW (depending on the pumping configurations and wavelength) that means the gain coefficient (also known as gain efficiency) being close to 0.06 dB/mW^12–14^. The efficiency optimization of an optical amplifier based on an active fiber includes two complementary approaches: confinement of doping area in a central part of an active fiber core and reducing the mode field size. In the case of bismuth-doped fibers, the former approach is difficult to realize due to the high volatility of bismuth atoms that leads to the bismuth-depleted glass in the center of a fiber core. The mode field size of an active fiber can be reduced by the management of the waveguide parameters, in particular, by an increase of the numerical aperture. However, the $\Delta$n increase by an addition of phosphorus oxide in the glass core has a number of undesired side effects, which deteriorate parameters of Bi-doped fibers. Such problems could be overcome by using the depressed cladding design concept^15,16^. This concept involves the creation of a depressed index ring-shaped region between the core and the cladding. The depressed cladding region decreases the coupling between the optical powers in the core and the cladding that can be used to reduce the sensitivity of a fiber to macrobending loss. Therefore, by the variation of the parameters of the ring-shaped region, such as its geometry and $\Delta$n, one can create a fiber with a reduced mode-field diameter and low bending loss.

In paper^17^, we fabricated and investigated a depressed cladding Bi-doped phosphosilicate glass-core fiber with low macrobending loss as a promising gain medium for a compact O-band amplifier. In this paper, we report results regarding the characterization of the BDFA based on this type of fibers using various pumping configurations. The experimental data on the performance of the BDFA with the active fiber coiled onto a reel with a radius of 1.5 cm are presented. In addition, a compact optical module of the BDFA operating at $\lambda$ $=$ 1.32 $\upmu$m pumped by a single commercially available laser diode at 1.23 $\upmu$m with a maximum output power of 250 mW is proposed.

**Figure 1:**
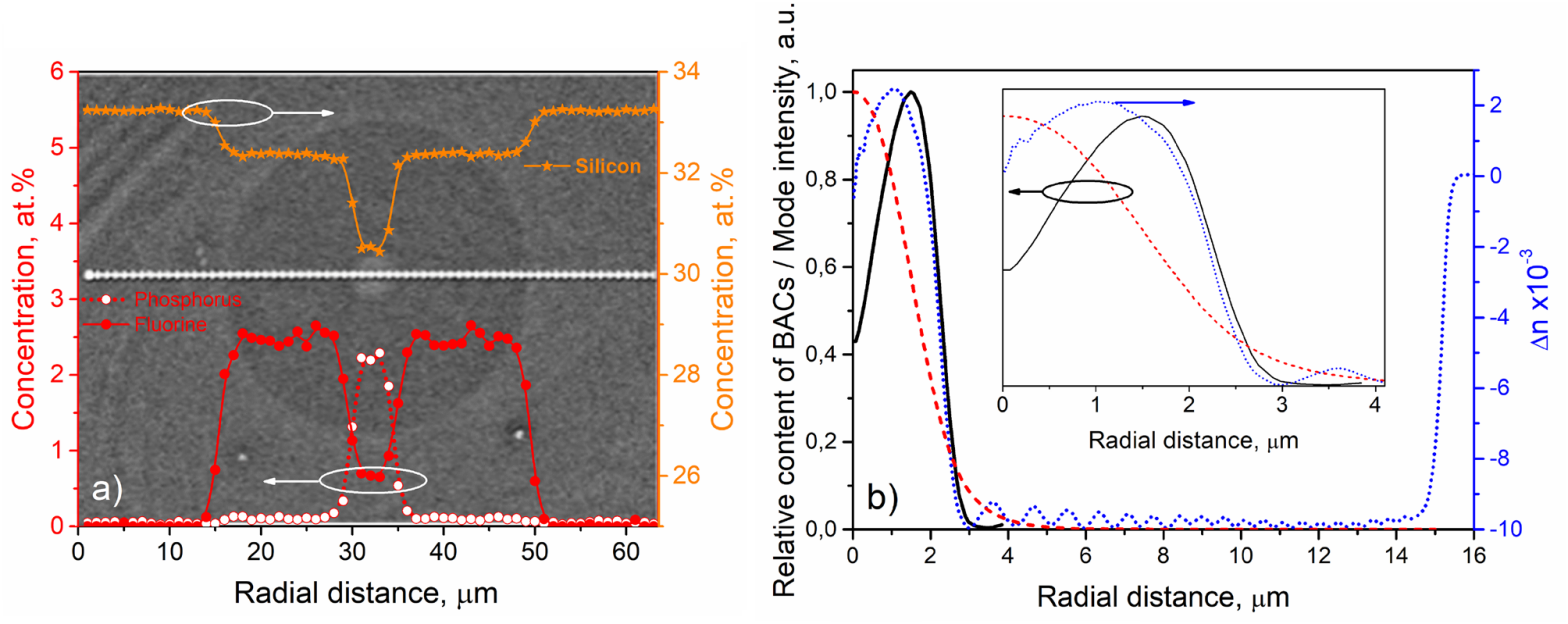
**(a)** Radial distributions of phosphorus, fluorine and silicon along a cross-section of the Bi-doped fiber where photography of the fiber endface is used as a background; the white-dotted trace corresponds to the EDX measurements; **(b)** solid line: distribution of the BACs (relative concentration, *n*(*r*)), dashed line: fundamental mode relative intensity profile (mode envelope, $\psi (r,\lambda )$), dotted line: refractive index difference profile. Inset: the same distributions within the core.

## Results and discussion

Figure 1a shows photography of an endface of a single-mode depressed cladding bismuth-doped phosphosilicate-core fiber. The fiber has the core and outer cladding diameters of 4.1 $\upmu$m and 125 $\upmu$m, respectively. The refractive index ($\Delta$n) difference profile of the fiber is presented in Fig. 1b. The index difference between the core and the first F-doped (depressed) cladding of the fiber was around 0.0115. The cutoff wavelength of the fiber was $\approx$1.0 $\upmu$m. The depressed cladding to the core radius ratio was about 6–7 as it is also clearly seen in Fig. 1a, where the radial distributions of Si, P and F concentrations along a cross-section of the studied fiber are presented. The measurement of radial distribution of the BACs in a fiber core is a rather complicated task due to the concentration being one or two orders of magnitude lower than the total Bi content, which was estimated to be 0.01 wt.$\%$. Moreover, the distribution of the BACs has no direct correlation to that of Bi ions because the formation of the BACs is also related to the structural features of the glass. Using the luminescence analysis, we measured the radial distribution of the BACs in the fiber preform and used these data in our calculation in the approximation of the similarity of the radial distribution of the BACs in preforms and the fibers drawn from these preforms. The obtained BACs distribution (to the fiber-core scale) is presented in Fig. 1b.

**Figure 2:**
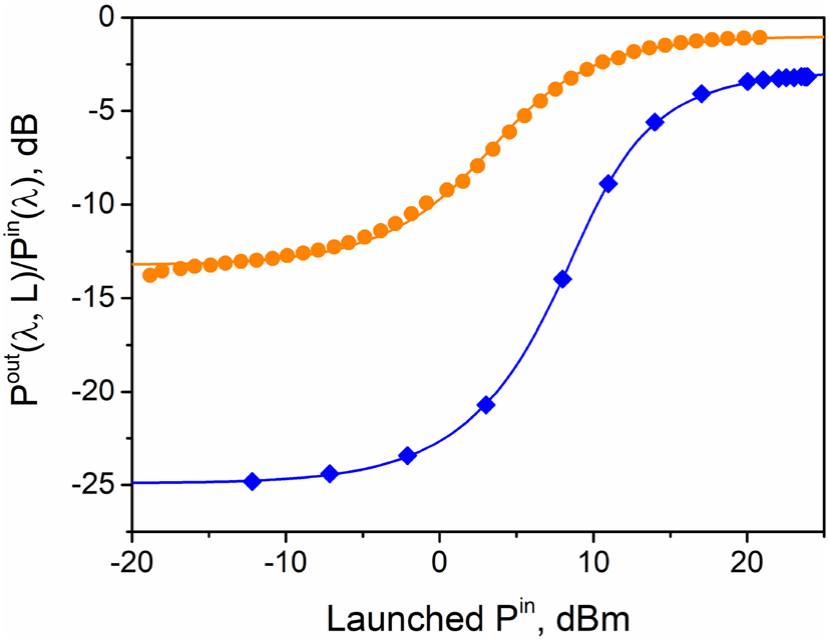
Transmission characteristics of the Bi-doped fiber as a function of launched power, used for the determination of the unsaturable loss and the saturation power. The solid curves correspond to the fitting of Eq. (1) to the experimental data designated by the markers (1230 nm—rhombus, 1310 nm—circles). The active fiber length *L* was equal to 30 m for the measurements at $\lambda _{s}$ $=$ 1310 nm and to 50 m for the measurements at $\lambda _{p}$ $=$ 1230 nm.

In our previous paper^17^, it was shown that this fiber has an excellent waveguide stability in the spectral region 1300–1400 nm even at a small bending radius of $\approx$1.5 cm. Thus, this approach can be used to produce a single-mode Bi-doped fiber with a smaller core diameter that is perspective for reducing the saturation power. We estimated the saturation powers $P_{sat}(\lambda )$ at the wavelengths of 1230 nm (pumping wavelength $\lambda _{p}$ ) and 1310 nm (a signal wavelength in the BDFA gain band $\lambda _{s}$ ) using the experimental data on the variation of the fiber transmission $T(\lambda , z) = \frac{P(\lambda , z)}{P^{in}(\lambda )}$ as a function of the launched pump power as shown in Fig. 2. In this case, we fitted the experimental data using Eq. (1) describing the propagation of the launched power $P(\lambda , z)$ along the longitudinal coordinate z taking into account the transverse variations of the intensity and the doping profile^18^.

1 $$\begin{aligned} \frac{d}{dz} \left\{ { Log \left[ \frac{P(\lambda , z)}{P^{in}(\lambda )}\right] } \right\} = - \alpha _{0}(\lambda )\cdot \frac{\int _{0}^{\infty }n(r)\cdot \psi _{s}(r, \lambda )\cdot \frac{1}{1+\frac{P(\lambda , z)}{P_{sat}(\lambda )}\cdot \psi _{s}(r, \lambda )}\cdot r dr}{\int _{0}^{\infty }n(r)\cdot \psi _{s}(r, \lambda )\cdot rdr}-\alpha _{BG}(\lambda ) \end{aligned}$$

where $P^{in}(\lambda )$ is the input power at wavelength $\lambda$; $\alpha _{0}(\lambda ) = 4.34 \times N_{0} \times \sigma _{a}(\lambda ) \times \Gamma (\lambda )$ is a small-signal absorption coefficient (maximum BACs concentration $\times$ absorption cross-section $\times$ power independent overlap integral); $\Gamma (\lambda ) = \frac{2}{\omega ^2}\cdot \int _{0}^{\infty }n(r)\cdot \psi _{s}(r, \lambda )\cdot rdr$, where $\omega ^2 = \int _{0}^{\infty } \psi _{s}(r, \lambda )\cdot rdr$ is, so-called, power radius; $n(r) = \frac{N(r)}{N_{0}}$ is a relative doping profile presented in Fig. 1b, where *N*(*r*) is the BACs distribution across the fiber transverse coordinate *r*^19^; $\psi _{s}$ is a mode envelope calculated with the OptiWave OptiFiber 2.0.2 software using the refractive index profile in Fig. 1b; $P_{sat}(\lambda )$ is the saturation power; and $\alpha _{BG}(\lambda )$ is the background (unsaturable) loss. The fixed and best-fit adjustable parameters of the fitting procedure are listed in Table 1.

**Table 1:** The fixed and best-fit adjustable parameters of the fitting of Eq. (1) to the experimental data on the variation of the fiber transmission with respect to the launched pump power at $\lambda _{p}$ and $\lambda _{s}$.

Designation	$\lambda _{p}$ $=$ 1230 nm	$\lambda _{s}$ $=$ 1310 nm
**Fixed parameters**		
$\omega$, $\upmu$m	1.83	1.93
$\Gamma (\lambda )$	0.66	0.63
**Best-fit parameters**		
$\alpha _{0}(\lambda )$, dB/m	0.45	0.41
$\alpha _{BG}(\lambda )$, dB/m	0.06	0.03
$P_{sat}(\lambda )$, mW	0.9	0.5

As it was expected, the saturation powers at $\lambda _{p}$ and $\lambda _{s}$ are noticeably lower compared to those of the existing Bi-doped phosphosilicate fibers. The contribution of the unsaturable loss at the wavelengths $\lambda _{p}$ and $\lambda _{s}$ to the total loss was evaluated as $\approx$ 13$\%$ and $\approx$ 7$\%$, respectively. The calculations are in good agreement with the experimental data. The calculations thus suggest the possibility of creation of a BDFA with improved parameters.

**Figure 3:**
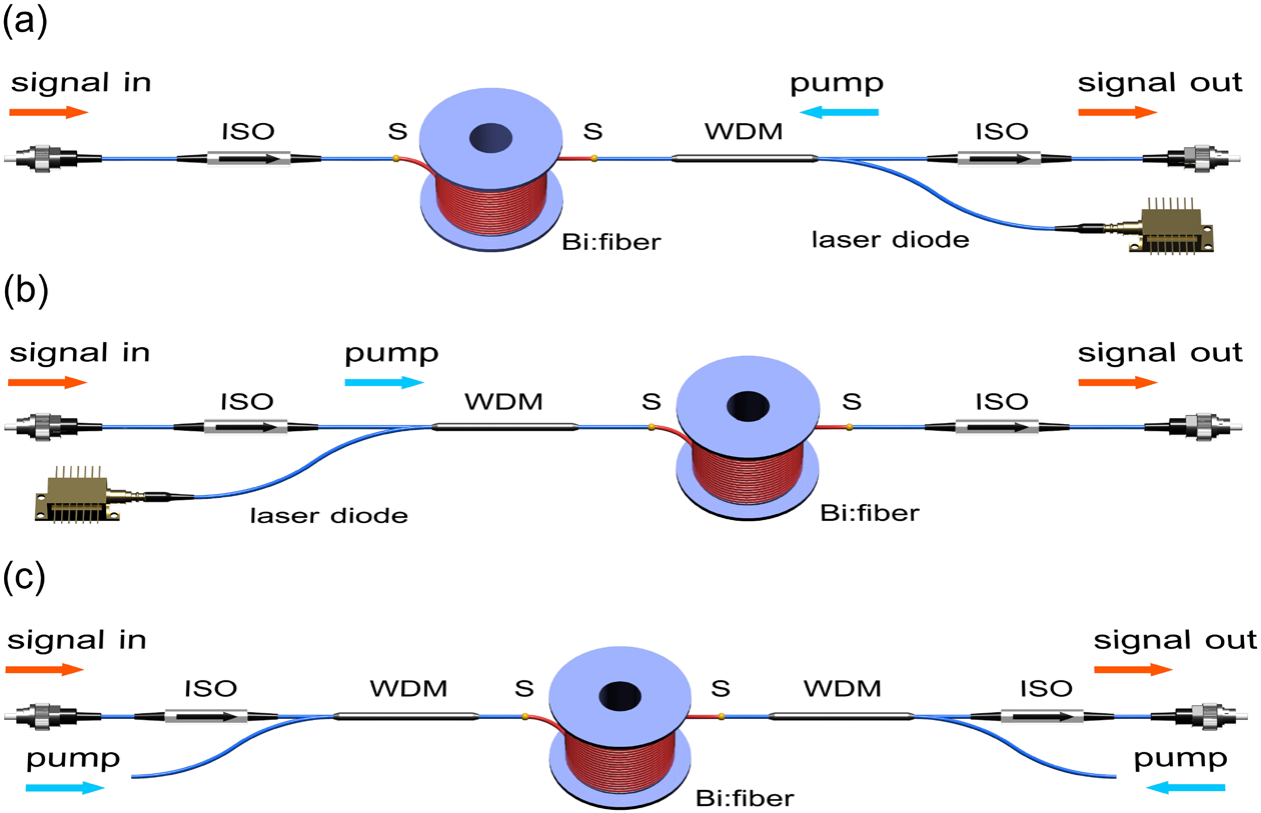
Basic configurations of the BDFA pumped by a laser diode operating at a wavelength of 1230 nm with: **(a)** unidirectional backward pumping; **(b)** unidirectional forward pumping and **(c)** bi-directional pumping. WDM—a wavelength division multiplexer; ISO—an optical isolator; S—splices of the active fiber with the optical components. The active fiber was wound on a spool with a radius of 1.5 cm. This figure was created by a free Blender 2.82a software (https://www.blender.org/).

As it has already been mentioned, in the case of compact packaging, the fiber macrobending loss could critically affect the performance of the fiber amplifier. It is caused not only by an increase of the background loss at the pump and signal wavelengths, but also by the outward displacement of the peak intensity of the mode from the fiber axis. Thus, the pump/signal mode could overlap noticeably worse with the Bi-doped region, thereby leading to the degradation of the BDFA gain performance. That is why we investigated the performance of the BDFA with the 140 m-long active fiber wound on a spool with a radius of $\approx$ 1.5 cm. The measurements of the gain and noise figure of the BDFA in the unidirectional (forward and backward) and bi-directional pumping configurations were carried out using the experimental setups shown in Fig. 3. All the experimental schemes of the BDFAs were constructed using commercially available fiber-optic components: fused fiber wavelength division multiplexers (WDMs) for coupling pump and signal powers (1230/1320 WDM Opto-Link Corporation); optical isolators (ISO) for pump and signal sources protection (S131-300-25-NC Opto-Link Corporation). Both a Raman fiber laser and a fiber-pigtailed laser diode operating at 1230 nm could be used as a pump source with an output power of 300 mW and more.

**Figure 4:**
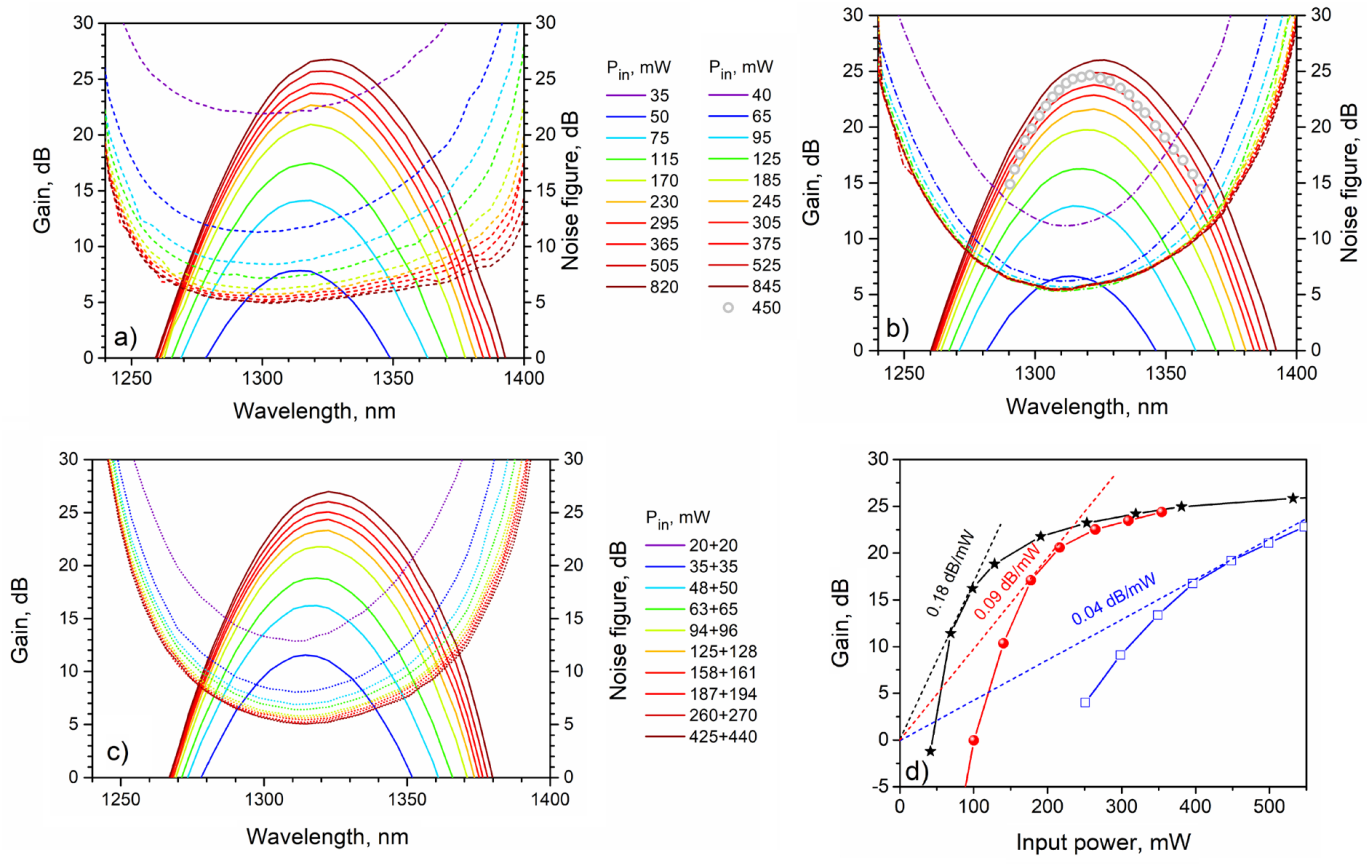
Gain (solid) and noise figure (dashed) spectra measured in the BDFA pumped near 1230 nm, for different input pump powers: **(a)** unidirectional backward pumping configuration, **(b)** unidirectional forward pumping configuration (circles show the data from Ref.^14^), **(c)** bi-directional pumping configuration; **(d)** Gain at 1.32 $\upmu$m as a function of the input pump power at 1.23 $\upmu$m (stars—the result of this work for the backward pumping configuration). The experimental data drawn from Refs.^12,14^, indicated by squares and balls, correspondingly, are also presented for comparison.

**Figure 5:**
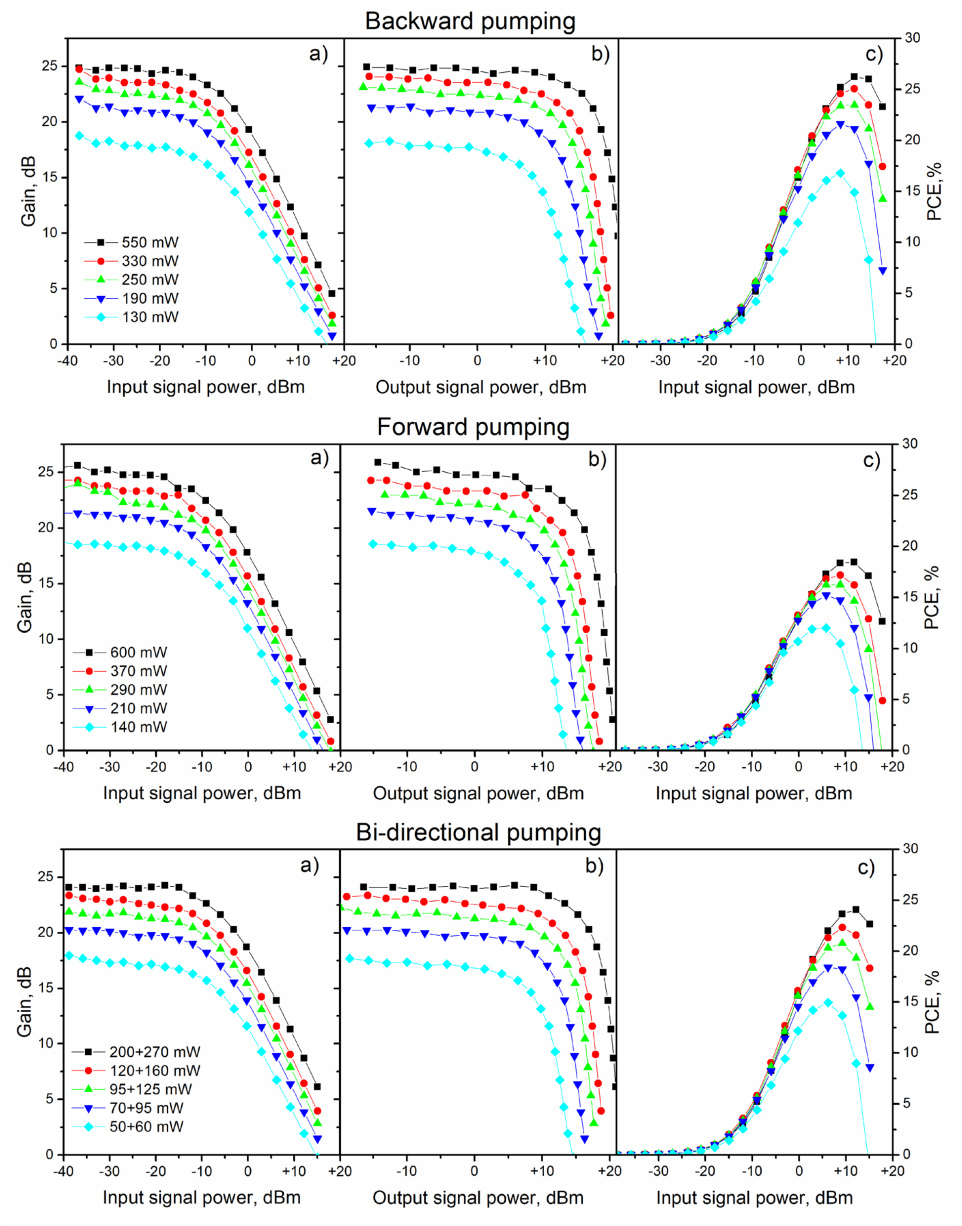
Gain at $\lambda$ = 1310 nm as a function of the input **(a)** and output **(b)** signal powers; **(c)** PCE versus the input signal power. Experimental data are presented for different configurations of the BDFA pumped at 1230 nm with different pump powers. The values of the input pump powers are presented in the corresponding configuration. For bi-directional pumping total (forward $+$ backward) pump powers are shown.

**Figure 6:**
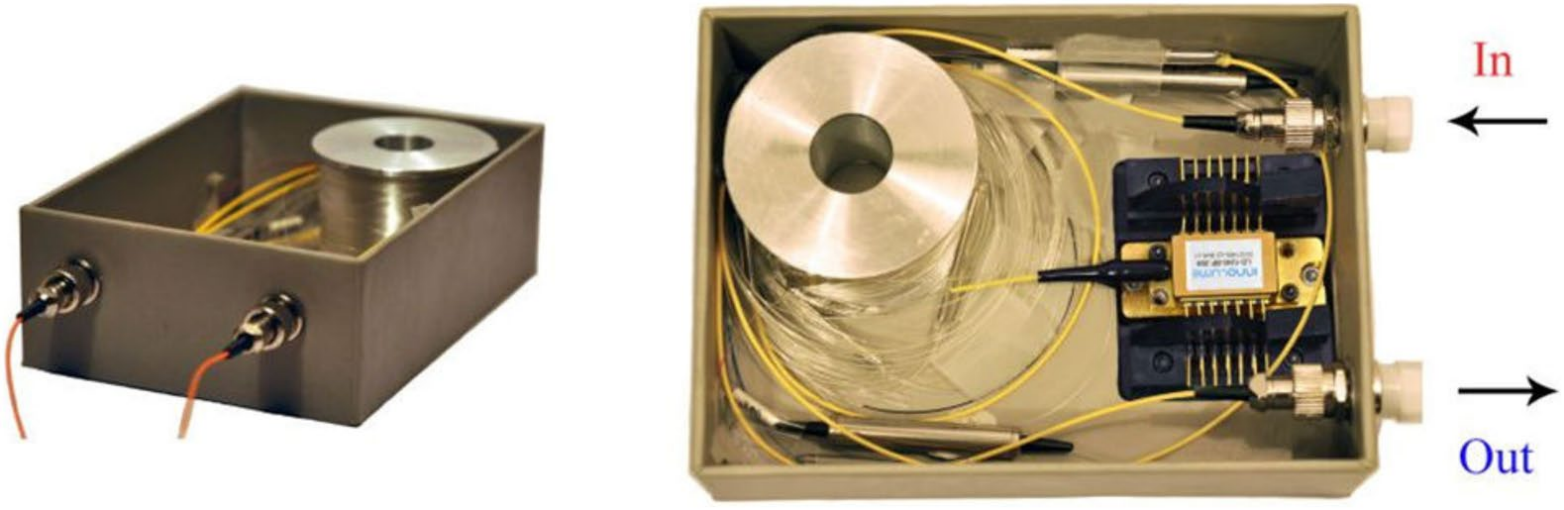
The optical module of the O-band BDFA constructed utilizing unidirectional backward pumping configuration using a commercially available laser diode operating at 1.23 $\upmu$m.

It should be noted that in the bi-directional pumping configuration, the pump power from a single pump source was divided with a fused 50/50 coupler that allowed us to obtain almost equal powers in each direction. A tunable light source was used as a signal source for the measurements of the gain and noise figure spectra. To obtain the signal power dependence of the BDFA characteristics, a home-built bismuth-doped fiber laser operating at 1.31 $\upmu$m was utilized. Figure 4 shows the gain and noise figure spectra for different input pump powers measured in various BDFA configurations pumped near 1230 nm. A probe signal power was $-25$ dBm at each wavelength with a spectral width of 4 nm. It is seen that peak gain values in the tested BDFAs are greater than 25 dB at a wavelength of 1320 nm with a noise figure of 5–6 dB. A gain value of 20 dB is achieved at the launched pump power of 250 mW that is almost two times lower compared to the earlier published data. A maximum width at a 3-dB level of the gain spectrum reached the value of $\approx$ 43 nm in unidirectional configuration of the BDFA. The shapes of the gain spectra obtained are similar to the best published results. This result, providing extremely important advantage of compactness, was expected because the effect of the macrobending loss in the active fiber is negligible as it was shown in^17^. In the case of the bi-directional pumping configuration, the gain spectrum is clearly narrower that is explained by the insertion loss due to an additional fused WDM. However, this problem can be solved by using filter WDMs (FWDMs) providing a broader transmission band. With an increase of pump power, one can observe the following changes: (i) spectral position of gain peak slightly shifts to the long-wavelength region regardless of the configuration of the BDFA; (ii) the noticeable broadening of the gain spectra; (iii) the noise figure values dropped to 5–6 dB. The observed shift of the maximum as well as the broadening of the gain spectra can be explained by a growth of the gain in the long-wavelength region attributed to another type of the BACs, namely, bismuth-related active centers associated with silicon, which are also formed in the fiber glass core^5^. In contrast to the gain spectra, the noise figure spectra significantly depend on the BDFA configurations. The configuration with unidirectional backward pumping provides the lowest noise figure 5–6 dB in the broad spectral region of 1270–1350 nm. In other configurations, 5–6 dB noise figures can be achieved only in the narrow wavelength bands ($\approx$ 1300–1325 nm for forward pumping and 1295–1335 nm for bi-directional pumping) as observed in Fig. 4a–c. This is a consequence of the insertion losses due to extra WDMs at the signal input. Fig. 4d presents the dependence of the gain on the input pump power for the backward pumping configuration. For the sake of comparison, we also illustrate the data published earlier for the optical amplifiers based on the bismuth-doped phosphosilicate fibers having step-index profiles. One can see that the gain coefficient (defined as gain per 1 mW of the input pump power in the small-signal regime) for the amplifier built in the present work turned out to be about 0.18 dB/mW that is greater than those of the Bi-doped fiber amplifiers fabricated so far.

In optical telecommunication systems, optical amplifiers can be used as boosters (power amplifiers), in-line and pre-amplifiers. Depending on the application, different requirements to the performance characteristics of amplifiers are to be established. For example, pre-amplifiers operating in small-signal regime have to provide high-level gain at low noise figure. At the same time, boosters are characterized by a high saturation output power while having relatively moderate gain and less strict requirements on the noise figure. In this regard, the study of the saturation effects in the BDFA is an important part of its characterization. Figure 5a, b presents the gain versus the input and output signal powers for different input pump powers. For the ease of comparison, all the data for different configurations of the BDFAs are presented in the same scale. It is seen that for the small-signal powers, signal gain is almost constant, however for the larger powers of the input signal gain saturation occurs. One can see a clear variation of the saturation input signal powers depending on the design of the BDFA. In particular, the saturation input signal power, determined as an input signal power at a 3-dB gain compression, is ranging from $-7$ to $-6$ dBm for different pumping schemes and almost independent of the input pump power (see Fig. 5a). As can be observed in Fig. 5b, the corresponding saturation output signal power does depend on the pump power and ranges from $+$ (7–8) to $+$ (14–16) dBm with the BDFA design. It should be noted that the maximum output signal power (defined as an output power at the gain equal to zero) is greater than $+$ 20 dBm (100 mW) for all the BDFA configurations. The dependencies of the pump-to-signal power conversion efficiency (PCE) of the BDFA on the input signal powers are presented in Fig. 5c. The backward configuration gives the highest PCE of above 27$\%$ since it maximizes the pump power in the region of the highest signal power. The lowest PCE of less than 20$\%$ is reached in the BDFA with forward configuration. The observed difference in terms of the PCE between the schemes can be attributed to the amplified spontaneous emission (ASE) related effects in the amplifier. For example, in the erbium-doped fiber amplifier, the generation of ASE is shown to perturb the pump distribution along a fiber, in a way that it is mostly detrimental to co-propagating (forward) configuration^18,20^. It should be noted that in the case of the BDFA the situation is further exacerbated by the fact that Bi-doped fibers have a much greater unsaturable loss compared with the Er-doped fibers.

Taking into account the results described above, one can conclude that the BDFAs can be potentially considered as compact all fiber devices (power booster or pre-amplifier) with high-performance characteristics constructed using only commercially available components. To accentuate this point, we also demonstrate a compact optical module of the BDFA operating in the spectral region near 1.3 $\upmu$m. Figure 6 shows photography of an optical BDFA module with the dimensions of 11.5 cm $\times$ 8 cm $\times$ 3.5 cm (L $\times$ W $\times$ H) which contains all the optical elements (two isolators, a spool with the active fiber, a fused WDM, a fiber-pigtailed semiconductor laser diode at 1230 nm with an output power of 300 mW) required for creation of the BDFA with unidirectional backward pumping configuration. It should be noted that this design does not contain the drive electronics for the pump diode which, as it is common, should be connected via an external interface. The proposed optical module is a first rough concept for the BDFA operating in the O-band and needs further improvements. But this is a purely engineering challenge which is beyond the scope of this paper.

## Conclusion

In conclusion, for the first time it has been shown that a depressed cladding bismuth-doped phosphosilicate fiber fabricated by the MCVD process is an appropriate gain medium for the creation of a compact and efficient all fiber optical amplifier operating in the spectral region near 1.3 $\upmu$m (O-band). We have developed and performed testing of a series of bismuth-doped fiber amplifiers having different pumping configurations where the active fiber was coiled on a reel with a radius of 1.5 cm. The performance characterization of these optical amplifiers pumped at a wavelength of 1230 nm with various pump powers was done at room temperature. We have obtained the dependencies of the gain on the input pump and signal powers, output powers and wavelengths, including those within the O-band. The record gain coefficient of 0.18 dB/mW (the pump-to-signal power conversion efficiency of above 27$\%$) has been achieved. In addition, a compact and efficient optical module for an O-band Bi-doped fiber amplifier constructed using only commercially available components has been demonstrated. We believe that the results of this research constitute an important step towards a widespread application of the bismuth-doped fiber amplifiers covering the O-band and will make possible to extend the capabilities of the conventional 1.3 $\upmu$m fiber-optic communication networks.

## Methods

A bismuth-doped phosphosilicate fiber preform was fabricated in-house using the conventional modified chemical vapor deposition (MCVD) technique. Detailed information about the fabrication process of this preform can be found in^17^. A single-mode bismuth-doped fiber was obtained from the fabricated preform by the drawing process with a rate of 10 m/min. The elemental analysis of the glass core of the investigated fiber with a spatial resolution of 1 $\upmu$m was performed by means of the Energy Dispersive X-Ray Spectroscopy (EDX) technique using a JSM-5910LV scanning electron microscope (JEOL) with an AZtecENERGY analytical system. Bismuth concentration of 0.01 wt.$\%$ was calculated from the experimental data obtained by the Electrothermal Atomization Atomic Absorption Spectrometry (EA-AAS) and the Inductively Coupled Plasma Atomic Emission Spectroscopy (ICP-AES). The small-signal absorption value at a wavelength of 1230 nm was determined from the absorption spectrum of the active fiber measured by the cut-back method using a halogen lamp and an optical spectrum analyzer (OSA). The unsaturable loss was derived from the experiments on a variation of the fiber transmission $T(\lambda , z) = \frac{P(\lambda , z)}{P^{in}(\lambda )}$ as a function of the launched pump power at the wavelengths of 1230 nm and 1310 nm. The radial BACs distribution in the preform of the investigated fiber was measured by means of the technique described in details in^19^. The parameters of the studied fiber were favorable for simple splicing with other optical fiber-pigtailed components by a splicer machine. The splicing losses were evaluated to be 0.15 dB. In all the cases, the small-signal gain coefficient at wavelength $\lambda$ was calculated by the relation of $G(\lambda ) = 10\cdot log(\frac{P_{s}^{out}(\lambda , P_{p}^{in})}{P_{s}^{in}(\lambda )})$ using the measured input $P_{s}^{in}(\lambda )$ and output $P_{s}^{out}(\lambda ,P_{p}^{in})$ powers of a narrow signal line. In the measurements of the gain and noise figure spectra, a signal wavelength was swept with a step of $\approx$4 nm. To calculate the noise figure of the BDFAs defined as $NF(\lambda ) = \frac{\rho _{ASE}\cdot \lambda }{G(\lambda )\cdot h \cdot c}$, (*c* is the speed of light in vacuum, *h* is the Plank’s constant) the ASE spectral density $\rho _{ASE}$ for different input pump powers were measured using the OSA. In the gain saturation experiments, to provide a variation of the signal power from a home-made bismuth-doped fiber laser at 1.31 $\upmu$m an optical attenuator was used. The pump-to-signal power conversion efficiency (PCE) was calculated according to the relation $PCE = \frac{P_{s}^{out}-P_{s}^{in}}{P_{p}^{in}}$ ($P_{s}^{out}, P_{s}^{in}$ are the output and input signal powers, respectively, $P_{p}^{in}$ is input pump power). The measurements of the pump and signal powers were provided by an Ophir powermeter with a 3FS detector. All the experiments were done at room temperature.
